# Identification and validation of the reference genes in the echiuran worm *Urechis unicinctus* based on transcriptome data

**DOI:** 10.1186/s12864-023-09358-6

**Published:** 2023-05-10

**Authors:** Jiao Chen, Yunjian Wang, Zhi Yang, Danwen Liu, Yao Jin, Xixi Li, Yuhang Deng, Boya Wang, Zhifeng Zhang, Yubin Ma

**Affiliations:** 1 Ministry of Education Key Laboratory of Marine Genetics and Breeding, College of Marine Life Sciences, Ocean University of China, Qingdao, China; 2 Key Laboratory of Tropical Aquatic Germplasm of Hainan Province, Sanya Oceanographic Institution, Ocean University of China, Sanya, China

**Keywords:** Reference genes, RT-qPCR, Transcriptome data, *Urechis unicinctus*

## Abstract

**Background:**

Real-time quantitative PCR (RT-qPCR) is a crucial and widely used method for gene expression analysis. Selecting suitable reference genes is extremely important for the accuracy of RT-qPCR results. Commonly used reference genes are not always stable in various organisms or under different environmental conditions. With the increasing application of high-throughput sequencing, transcriptome analysis has become an effective method for identifying novel stable reference genes.

**Results:**

In this study, we identified candidate reference genes based on transcriptome data covering embryos and larvae of early development, normal adult tissues, and the hindgut under sulfide stress using the coefficient of variation (CV) method in the echiuran *Urechis unicinctus*, resulting in 6834 (15.82%), 7110 (16.85%) and 13880 (35.87%) candidate reference genes, respectively. Gene Ontology (GO) and Kyoto Encyclopedia of Genes and Genomes (KEGG) enrichment analyses revealed that the candidate reference genes were significantly enriched in cellular metabolic process, protein metabolic process and ribosome in early development and normal adult tissues as well as in cellular localization and endocytosis in the hindgut under sulfide stress. Subsequently, ten genes including five new candidate reference genes and five commonly used reference genes, were validated by RT-qPCR. The expression stability of the ten genes was analyzed using four methods (geNorm, NormFinder, BestKeeper, and ∆Ct). The comprehensive results indicated that the new candidate reference genes were more stable than most commonly used reference genes. The commonly used *ACTB* was the most unstable gene. The candidate reference genes *STX12*, *EHMT1*, and *LYAG* were the most stable genes in early development, normal adult tissues, and hindgut under sulfide stress, respectively. The log_2_(TPM) of the transcriptome data was significantly negatively correlated with the Ct values of RT-qPCR (Ct =  − 0.5405 log_2_(TPM) + 34.51), which made it possible to estimate the Ct value before RT-qPCR using transcriptome data.

**Conclusion:**

Our study is the first to select reference genes for RT-qPCR from transcriptome data in Echiura and provides important information for future gene expression studies in *U. unicinctus*.

**Supplementary Information:**

The online version contains supplementary material available at 10.1186/s12864-023-09358-6.

## Background

Real-time quantitative PCR (RT-qPCR) is the most widely used technique for relative gene quantification because of its good repeatability, high sensitivity, strong specificity, high throughput, simplicity, speed, and low cost [1–3]. However, the biological variability of initial materials and the technical factors involved in sample preparation, such as the quantity of cDNA, RNA extraction, RNA integrity, and storage conditions will inevitably affect the accuracy of RT-qPCR [4–6]. Therefore, normalization is necessary to correct for variations in template quantity. Reference genes are used for the normalization of gene expression because of the stability of expression levels among different tissues, different developmental stages, or under various treatments [5].

In general, constitutively expressed housekeeping genes are used as reference genes, such as actin, elongation factor, glyceraldehyde-3-phosphate dehydrogenase, ribosomal RNA, translation initiation factor, tubulin, and ubiquitin [7–9]. However, many studies have shown that some of these genes are not always stable and their expression levels vary greatly under specific experimental conditions [6, 10, 11]. This is especially true for non-model organisms, which currently lag behind well-characterized model organisms in terms of genomic resources and empirically tested reference genes [4, 6, 10, 11]. Moreover, recent studies have shown that it is impossible to totally normalize gene expression data from all sample types using a single gene [6, 10]. Therefore, two or more reference genes are desirable to improve the reliability and accuracy of the RT-qPCR results. With the increasing application of high-throughput sequencing, RNA-seq has provided a new strategy for identifying new highly stable reference genes from transcriptome data. Heretofore, identification of many novel reference genes has been performed based on transcriptome data in various organisms [10–22].

The Echiura worm *Urechis unicinctus*, a typical benthic species living in intertidal sediments, is widely distributed in Russia, Korean Peninsula, Japan and China [23]. *U. unicinctus* possesses high economic value because of great edible value and potential medical value [23]. *U. unicinctus* is also mostly used to study gametogenesis [24], development [25–31], evolution [32, 33], and sulfide metabolism [34] because of its characteristics such as a large number of eggs laid, high fertilization rate, biphasic life cycle, and high sulfide tolerance ability. Recently, the use of *U. unicinctus* in evo-devo studies has generated many breakthrough [32, 33]. Evolutionary transcriptome analysis of the trochophores of *U. unicinctus* and other metazoan animals reveal an adult-first evolutionary scenario with a single metazoan larval intercalation [32]. Hox-mediated body plan diversification is an important developmental process [35]. In *U. unicinctus*, the expression of *Hox* genes exhibits a subcluster-based whole-cluster spatio-temporal collinearity pattern, suggesting that *Hox* subcluster play an important role in spatio-temporal collinearity pattern in invertebrates [33]. On the other hand, as a species living in the intertidal zone, *U. unicinctus* can tolerate, metabolize and utilize environmental sulfide and is considered a model species for sulfide adaptation [34, 36–45].

At present, gene expression analysis by RT-qPCR has been widely performed in *U. unicinctus*, using commonly used reference genes, such as *ATPase* [29–31, 33, 46] and *β-actin* [34, 36, 40, 41, 43, 47–51]. Previous studies have identified some reference genes, but have generally focused on some traditionally used genes, such as *EF-1-α*, *TBP*, *TUB*, *eIF3*, and *ATPase* [52, 53]. Suitable reference genes are crucial for verifying the expression profiles of related genes for future studies in *U. unicinctus*. RNA-Seq, which can provide a large amount of gene transcription information, is a better method for reference gene screening [10–22]. In addition to classical housekeeping genes, transcriptome data analysis provides an opportunity to identify novel and more stable reference genes. In recent years, a large amount of transcriptome data of *U. unicinctus* has been published [32, 45, 46, 54], which provides a new strategy for selecting housekeeping genes or reference genes in *U. unicinctus*.

In this study, we systematically screened reference genes by analyzing transcriptome data including early development, normal adult tissues, and the hindgut under sulfide stress in *U. unicinctus*. Candidate reference genes were selected from the three datasets. Moreover, the correlation between the Ct of RT-qPCR and transcripts per million (TPM) of the transcriptome data was investigated. Our findings identified novel stable reference genes from transcriptome data and contributed to the accurate quantification of gene expression in *U. unicinctus*.

## Results and discussion

### Identification of the candidate reference genes from transcriptome data

**Fig. 1:**
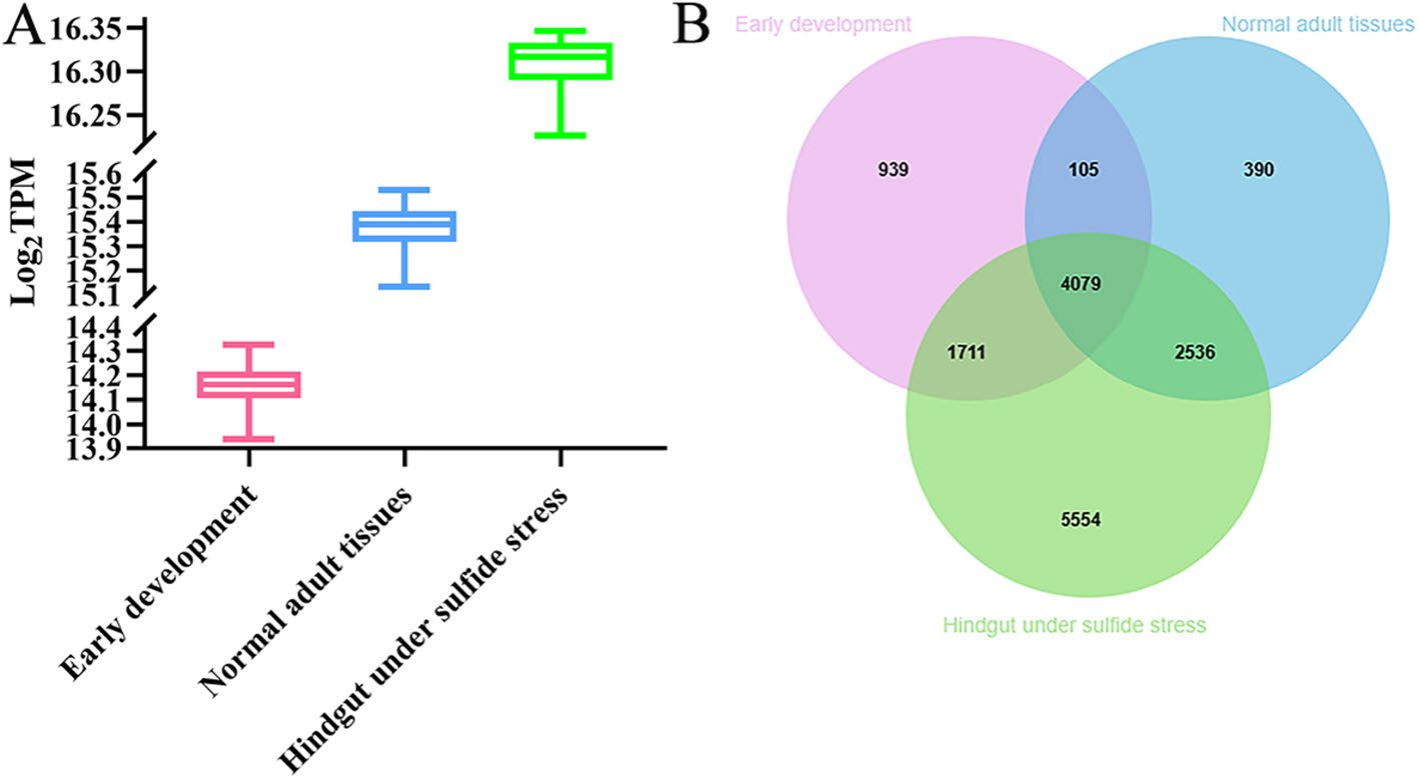
The selected candidate reference genes in early development, normal adult tissues, and the hindgut under sulfide stress. **A** Boxplot exhibiting the log_2_TPM values of candidate reference genes that met the criteria. **B** Venn diagram showing the relationships between candidate reference genes under three conditions. Pink, early development; blue, normal adult tissues; green, hindgut under sulfide stress

In this study, we systematically screened reference genes based on transcriptome datasets from early developmental embryos and larvae, normal adult tissues, and the hindgut under sulfide stress using the coefficient of variation (CV) method in *U. unicinctus*. The CV method is simple to use for candidate reference gene selection. Moreover, compared with other methods such as the fold change method, the CV method can quantify expression variability in a way in which genes can be ranked and directly compared, which has previously been used to identify novel reference genes from transcriptome data in plant species such as the monkeyflower genera *Mimulus luteus*, *Polygonum cuspidatum*, apple, and *Lycium barbarum* L [12, 16, 21, 55] and animals such as *Mizuhopecten yessoensis*, and silkworm *Bombyx mori* [17, 18]. Although both reads/fragments per kilobase per million (RPKM or FPKM) and TPM can be used to measure gene expression levels, RPKM and FPKM may not be applicable to the comparison of gene expression levels because of the differences sequencing depth between samples. TPM is more suitable for comparison of expression levels among samples [11, 56]. First, we excluded genes with low expression levels for easy detection in RT-qPCR assays and adopted a minimum mean log_2_(TPM) cut-off of 5 as a criterion for gene expression levels. Second, to ensure that the reference genes had low variance, a standard deviation (SD) log_2_(TPM) value of less than 1 was required. So a 0.2 CV cut-off was applied to further identify reference genes, which has been recommended in previous studies [11]. Based on these criteria, we identified 6834 (15.82%), 7110 (16.85%) and 13880 (35.87%) candidate reference genes from 43209 genes of early developmental embryos and larvae, 42191 genes of different normal adult tissues, and 38690 genes of the hindgut under sulfide stress. The number of candidate reference genes for early development was lowest. This result was expected because gene expression levels can change dramatically in a short time during early development [11]. Further, the expression levels of the candidate reference genes were analyzed. The results indicated that the median log_2_(TPM) values of the candidate reference genes were 14.162 in the early developmental stages, 15.389 in normal adult tissues and 16.317 in hindgut under sulfide stress (Fig. 1A). The ten most stable genes with the lowest CV values in early developmental embryos and larvae, different normal adult tissues, and hindgut under sulfide stress are listed in Table 1. The mean log_2_(TPM) and CV values of the ten most stable genes in the early development stages ranged from 14.317 to 14.329 and 0.0157 to 0.0203, respectively. All genes were annotated, of which six encoded proteins (*FXRD1*, *IF2M*, *STX12*, *PTCD3*, *TCF25*, and *TFG*) related to gene transcription, translation, protein transport and assembly, and three encoded proteins (*CAPR1*, *NSMA*, *PDCD6*, and *HBS1L*) related to cell growth, proliferation and apoptosis. As for the ten most stable genes in normal adult tissues, their mean log_2_(TPM) and CV values ranged from 15.524 to 15.532 and 0.0093 to 0.0138, respectively. Nine genes were annotated, of which six were encoded proteins (*UBE4A*, *OGT1*, *GGA3*, *EHMT1*, *TRA2B*, and *PRP39*) related to protein modification, transport and mRNA splicing. The ten most stable genes in the hindgut under sulfide stress showed mean log_2_(TPM) values ranged from 16.3457 to 16.3463 and CV values ranging from 0.0018 to 0.0032. Nine genes were annotated, of which three (*CNOT1*, *RBM26*, and *HTR5B*) encoded proteins related to post-transcriptional regulation.

**Table 1:** The information of the top 10 candidate reference genes in the early development, normal adult tissues and hindgut under sulfide stress of the *U. unicinctus*

	Gene ID	Gene name	CV	Mean
Early development	evm.model.Hic_asm_9.985.3	FAD-dependent oxidoreductase domain-containing protein 1, *FXRD1*	0.0157	14.329
	evm.model.Hic_asm_2.658	Caprin-1, *CAPR1*	0.0170	14.326
	evm.model.Hic_asm_11.431	Putative neutral sphinGOmyelinase, *NSMA*	0.0171	14.326
	evm.model.Hic_asm_13.131	Translation initiation factor IF-2 Mitochondrial, *IF2M*	0.0183	14.323
	evm.model.Hic_asm_2.533	Syntaxin-12, *STX12*	0.0190	14.322
	evm.model.Hic_asm_6.652	Programmed cell death protein 6, *PDCD6*	0.0191	14.326
	evm.model.Hic_asm_3.310.1	Pentatricopeptide repeat domain-containing protein 3, mitochondrial, *PTCD3*	0.0192	14.320
	evm.model.Hic_asm_6.1042	Transcription factor 25, *TCF25*	0.0201	14.319
	evm.model.Hic_asm_11.1625	TRK-fused gene protein*, TFG*	0.0202	14.319
	evm.model.Hic_asm_0.1156	HBS1-like protein, *HBS1L*	0.0203	14.317
Normal adult tissues	evm.model.Scaf262.4	Ubiquitin conjugation factor E4 A, *UBE4A*	0.0093	15.532
	evm.model.Hic_asm_10.1197	UDP-N-acetylglucosamine–peptide N-acetylglucosaminyltransferase 110 kDa subunit, *OGT1*	0.0102	15.531
	novel.9221	Not annotated	0.0114	15.528
	evm.model.Hic_asm_9.1113_evm.model.Hic_asm_9.1114	Histone-lysine N-methyltransferase, *EHMT1*	0.0114	15.528
	evm.model.Hic_asm_8.1166	ADP-ribosylation factor-binding protein, *GGA3*	0.0118	15.528
	evm.model.Hic_asm_7.291	Transformer-2 protein homolog beta, *TRA2B*	0.0125	15.526
	evm.model.Hic_asm_4.469	F-box only protein 18, *FBX18*	0.0129	15.525
	evm.model.Hic_asm_3.391	Uncharacterized protein STK_23830	0.0131	15.525
	evm.model.Hic_asm_13.300	Pre-mRNA-processing factor 39, *PRP39*	0.0134	15.524
	evm.model.Scaf156.13	Rabenosyn-5, *RBNS5*	0.0138	15.524
Hindgut under sulfide stress	evm.model.Hic_asm_6.1089	CCR4-NOT transcription complex subunit 1, *CNOT1*	0.0018	16.346
	evm.model.Hic_asm_1.1227	Clathrin heavy chain 1, *CLH1*	0.0027	16.346
	evm.model.Hic_asm_3.989	Exocyst complex component 6, *EXOC6*	0.0027	16.346
	evm.model.Hic_asm_14.1611	Lysosomal alpha-glucosidase, *LYAG*	0.0027	16.346
	evm.model.Hic_asm_7.654	Not annotated	0.0028	16.346
	evm.model.Hic_asm_3.766	Melanotransferrin, *TRFM*	0.0030	16.346
	evm.model.Hic_asm_16.105	Angiomotin, *AMOT*	0.0031	16.346
	evm.model.Hic_asm_10.1107	RNA-binding protein 26, *RBM26*	0.0031	16.346
	evm.model.Hic_asm_2.1309	Calumenin-B, *CALUB*	0.0031	16.346
	evm.model.Hic_asm_13.2016	HEAT repeat-containing protein 5B, *HTR5B*	0.0032	16.346

### Functional enrichment analysis of candidate reference genes

To further analyze the relationships of candidate reference genes in early development, normal adult tissues, and the hindgut under sulfide stress, we compared the three candidate reference gene datasets. As shown in Fig. 1B, 4079 genes were shared in three candidate reference gene datasets, 4184 genes were shared in early development and normal adult tissues, 5790 genes were shared in early development and hindgut under sulfide stress, and 6615 genes were shared in normal adult tissues and hindgut under sulfide stress. Gene Ontology (GO) and Kyoto Encyclopedia of Genes and Genomes (KEGG) enrichment analyses of the three candidate reference gene datasets were then performed (Table 2). In early development, GO enrichment analysis showed that the candidate reference genes were mainly enriched in biological process (BP) terms associated with cellular protein metabolic process and macromolecule metabolic process, and in molecular function (MF) terms related to binding. These genes were also enriched in cellular component (CC) terms associated with intracellular and cell. KEGG pathway enrichment analysis indicated ribosome was the most significant pathway, followed by proteasome. In different adult tissues, GO enrichment analysis showed that the candidate reference genes were mainly enriched in biological process (BP) terms associated with cellular protein metabolic process and intracellular transport, and in molecular function (MF) terms related to binding. These genes were also enriched in cellular component (CC) terms associated with intracellular and cell. KEGG pathway enrichment analysis indicated ribosome was the most significant pathway, followed by oxidative phosphorylation. In the hindgut under sulfide stress, GO enrichment analysis showed that the candidate reference genes were mainly enriched in biological process (BP) terms associated with protein phosphorylation and in molecular function (MF) terms related to protein binding. These genes were also enriched in cellular component (CC) terms associated with intracellular, cell and organelle. KEGG pathway enrichment analysis revealed diabetic cardiomyopathy was the most significant pathway, followed by the oxidative phosphorylation. In summary, the results of GO and KEGG enrichment analysis in the hindgut under sulfide stress were different from those in early development and different normal adult tissues, with the proportion of specific candidate reference genes belonging to the hindgut under sulfide stress being the largest, suggesting that the hindgut under sulfide stress, early developmental embryos and larvae, and different adult tissues may focus on diverse biological processes or pathways. Therefore, we need to screen for optimal reference genes in early development, different normal adult tissues, and the hindgut under sulfide stress.

**Table 2:** GO and KEGG pathways enriched in the candidate reference genes

	ID code	Term/ Pathway	Subgroup	Gene number	Q value
Early development	GO				
	GO:0044267	cellular protein metabolic process	Biological process	679 (19.73%)	0.000000
	GO:0044260	cellular macromolecule metabolic process	Biological process	1518 (44.1%)	0.000000
	GO:0044237	cellular metabolic process	Biological process	1883 (54.71%)	0.000000
	GO:0046907	intracellular transport	Biological process	281 (8.16%)	0.000000
	GO:0005622	intracellular	Cellular component	1315 (57%)	0.000000
	GO:0005623	cell	Cellular component	1414 (61.29%)	0.000000
	GO:0044464	Cell part	Cellular component	1414 (61.29%)	0.000000
	GO:0044424	intracellular part	Cellular component	1263 (54.75%)	0.000000
	GO:0043226	organelle	Cellular component	1032 (44.73%)	0.000000
	GO:0043229	intracellular organelle	Cellular component	1014 (43.95%)	0.000000
	GO:0005488	binding	Molecular function	3127 (72.4%)	0.000000
	GO:0005515	protein binding	Molecular function	1612 (37.32%)	0.000000
	GO:0043168	anion binding	Molecular function	753 (17.43%)	0.000000
	GO:0000166	nucleotide binding	Molecular function	737 (17.06%)	0.000000
	KEGG				
	ko03010	Ribosome		89 (4.38%)	0.000000
	ko03050	Proteasome		37 (1.82%)	0.000000
	ko04141	Protein processing in endoplasmic reticulum		97 (4.77%)	0.000000
	ko04120	Ubiquitin mediated proteolysis		84 (4.13%)	0.000001
	ko03420	Nucleotide excision repair		33 (1.62%)	0.000002
	ko05171	Coronavirus disease—COVID-19		75 (3.69%)	0.000010
Normal adult tissues	GO				
	GO:0044267	cellular protein metabolic process	Biological process	743 (20.74%)	0.000000
	GO:0046907	intracellular transport	Biological process	298 (8.32%)	0.000000
	GO:0051649	establishment of localization in cell	Biological process	323 (9.01%)	0.000000
	GO:0044260	cellular macromolecule metabolic process	Biological process	1577 (44.01%)	0.000000
	GO:0005622	intracellular	Cellular component	1364 (57.97%)	0.000000
	GO:0044424	intracellular part	Cellular component	1312 (55.76%)	0.000000
	GO:0005623	cell	Cellular component	1458 (61.96%)	0.000000
	GO:0044464	cell part	Cellular component	1458 (61.96%)	0.000000
	GO:0043226	organelle	Cellular component	1084 (46.07%)	0.000000
	GO:0,043,229	intracellular organelle	Cellular component	1056 (44.88%)	0.000000
	GO:0005488	bind	Molecular function	3371 (74.51%)	0.000000
	GO:0005515	protein binding	Molecular function	1763 (38.97%)	0.000000
	GO:0043168	anion binding	Molecular function	801 (17.71%)	0.000000
	GO:0017076	purine nucleotide binding	Molecular function	668 (14.77%)	0.000000
	KEGG				
	ko03010	Ribosome		96 (5.02%)	0.000000
	ko00190	Oxidative phosphorylation		67 (3.51%)	0.000000
	ko03050	Proteasome		38 (1.99%)	0.000000
	ko05171	Coronavirus disease—COVID-19		85 (4.45%)	0.000000
	ko05415	Diabetic cardiomyopathy		85 (4.45%)	0.000000
	ko05016	Huntington disease		141 (7.38%)	0.000000
Hindgut under sulfide stress	GO				
	GO:0006468	protein phosphorylation	Biological process	329 (4.82%)	0.000000
	GO:0007264	small GTPase mediated signal transduction	Biological process	225 (3.3%)	0.000000
	GO:0051641	cellular localization	Biological process	558 (8.18%)	0.000000
	GO:0051649	establishment of localization in cell	Biological process	513 (7.52%)	0.000000
	GO:0005622	intracellular	Cellular component	2248 (48.43%)	0.000000
	GO:0005623	cell	Cellular component	2461 (53.02%)	0.000000
	GO:0044464	cell part	Cellular component	2461 (53.02%)	0.000000
	GO:0044424	intracellular part	Cellular component	2147 (46.25%)	0.000000
	GO:0043226	organelle	Cellular component	1776 (38.26%)	0.000000
	GO:0043229	intracellular organelle	Cellular component	1729 (37.25%)	0.000000
	GO:0005515	protein binding	Molecular function	3325 (38.6%)	0.000000
	GO:0005488	binding	Molecular function	6141 (71.3%)	0.000000
	GO:0043168	anion binding	Molecular function	1372 (15.93%)	0.000000
	GO:0017076	purine nucleotide binding	Molecular function	1119 (12.99%)	0.000000
	KEGG				
	ko05415	Diabetic cardiomyopathy		102 (3.23%)	0.000004
	ko00190	Oxidative phosphorylation		73 (2.31%)	0.000004
	ko05017	Spinocerebellar ataxia		97 (3.07%)	0.000008
	ko03050	Proteasome		38 (1.2%)	0.000013
	ko04136	Autophagy—other		36 (1.14%)	0.000026
	ko04120	Ubiquitin mediated proteolysis		107 (3.39%)	0.000026

### Validation of candidate and commonly used reference genes expression stability by RT-qPCR assay

Five candidates and five commonly used reference genes were selected for validation and comparison of the expression stability in this study (Additional file 1: Table S1). Five of the top ten candidate reference genes were chosen for RT-qPCR in each case, as shown in Table 1. The five candidate reference genes were *FXRD1*, *CAPR1*, *NSMA*, *IF2M*, and *STX12* in early development; *UBE4A*, *OGT1*, *EHMT1*, *GGA3*, and *TRA2B* in normal adult tissues and *CNOT1*, *CLH1*, *EXOC6*, *LYAG*, and *TRFM* in the hindgut under sulfide stress. The five commonly used reference genes were *ATPase B*, *TBP*, *eIF3*, *ACTB*, and *GAPDH* (Table 3).

**Table 3:** Detailed information on the five commonly used reference genes in the early development, normal adult tissues, and the hindgut under sulfide stress of the *U. unicinctus*

GenBank ID	Gene name	Early development		Normal adult tissues		Hindgut under sulfide stress	
		CV	Mean	CV	Mean	CV	Mean
MN662252	*ATPase B*	0.0623	14.128	0.0424	15.415	0.0150	16.327
OQ111925	*TBP*	0.0956	13.568	0.0360	15.435	0.0154	16.325
MN662253	*eIF3*	0.0517	14.205	0.0495	15.361	0.0131	16.331
GU592178	*ACTB*	0.2601	12.530	0.1265	14.444	0.0132	16.330
EF012775	*GAPDH*	0.0887	13.880	0.0607	15.235	0.0202	16.312

**Fig. 2:**
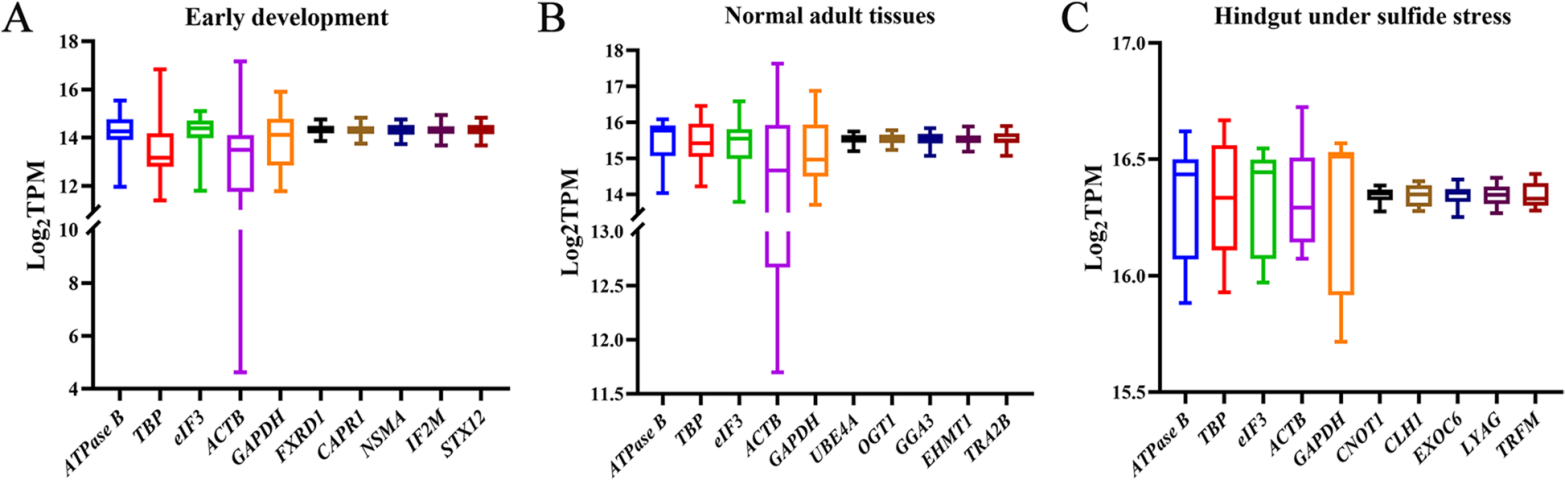
The boxplots that show the log_2_TPM values of the five commonly used reference genes and five candidate reference genes in early development (**A**), normal adult tissues (**B**) and hindgut under sulfide stress

Boxplots were constructed to present the expression levels of five candidate reference genes and five commonly used reference genes under all three conditions (Fig. 2). As shown in Fig. 2, Table 1, and Table 3, the novel candidate reference genes possessed higher stability than the commonly used reference genes in all three conditions. The variances in the commonly used reference genes were different among three cases. Three of the five commonly used reference genes, *ACTB*, *GAPDH*, and *TBP* are unstable during early development. *ACTB* was the most unstable reference gene in early development and normal adult tissues. *GAPDH* have the highest variance in the hindgut under sulfide stress, followed by *TBP*.

**Fig. 3:**
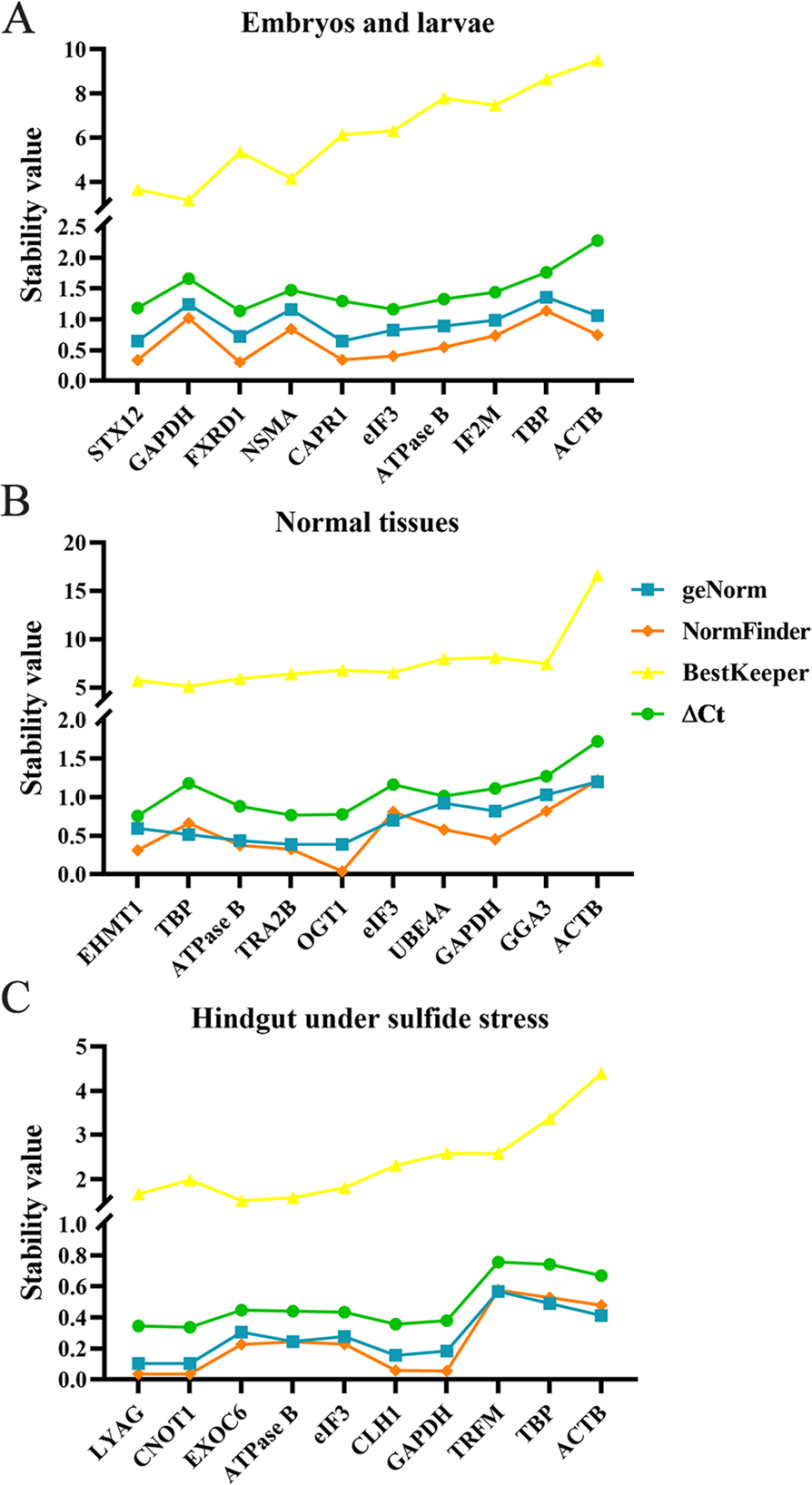
Expression stability of the ten genes in early development (**A**), normal adult tissues (**B**), and hindgut under sulfide stress (**C**). Stability was estimated using geNorm, NormFinder, BestKeeper, and ∆Ct analyses of the RT-qPCR data. The genes were ranked in descending order of expression stability from left to right after comprehensive analysis

To further examine the results of transcriptome analysis, RT-qPCR experiments were carried out, and the expression level constancy of ten reference genes in different cases was assessed by four data processing methods (geNorm, NormFinder, BestKeeper, and ∆Ct) [57–60]. Despite a slight difference in the samples used between transcriptome data and RT-qPCR, the results of transcriptome analysis and RT-qPCR assay are very similar, which suggests that candidate reference genes have higher stability than most of the commonly used genes. As shown in Fig. 3A, during early development, syntaxin-12 (*STX12*) was the most stable gene, which is a member of the syntaxin family localized to the endosome [61]. The syntaxin family belongs to the t-SNARE subfamily of the SNARE superfamily and is involved in vesicle trafficking [62, 63]. *STX12* is widely expressed and potentially participates in a common trafficking event that occurs in every cell [64–66], which explains why *STX12* showed stable expression levels during early development. The most stable reference gene in different adult tissues was euchromatic histone-lysine N-methyltransferase 1 (*EHMT1*) (Fig. 3B). EHMT1 and euchromatic histone-lysine N-methyltransferase 2 (EHMT2) are highly homologous and generate functional heterodimeric complexes that are mainly responsible for mono- and dimethylation of histone H3 lysine 9 (H3K9) in euchromatin [67]. EHMT1/EHMT2 is essential for maintaining the normal methylation patterns of H3K9 and plays a central role in the epigenetic control of euchromatin, which is vital for normal cell function. They are universally expressed and associated with many biological processes [67–71]. EHMT1 is also required for normal levels of DNA methylation in facultative heterochromatin [72]. The stable expression levels of *EHMT1* and its key role in cells enabled its use as a reference gene in normal adult tissues. As to hindgut under sulfide stress, the most stable gene was lysosomal alpha-glucosidase (*LYAG*) (Fig. 3C), which is a retaining exo-glucosidase catalyzing the production of glucose from glycogen in lysosomes [73, 74]. LYAG is extremely important for the degradation of glycogen in lysosomes [75]. This defect can cause the substrate to accumulate in almost all body tissues [76]. Alpha-glucosidases have weak specificity and a given substrate is not strictly connected to a single type of protein [77]. The expression levels of *LYAG* were not affected by sulfide treatment. Therefore, *STX12*, *EHMT1* and *LYAG* can be selected as reference genes to normalize the results of the RT-qPCR assay in early development, normal adult tissues, and hindgut after sulfide stress in *U. unicinctus*, respectively.

Compared to the novel screened candidate reference genes, most of the commonly used reference genes had lower comprehensive ranking values. During early development, *ACTB* was the most variable, followed by *TBP*, which is consistent with the transcriptome data of the two genes with high CV values (Fig. 3A, Table 3). Similarly, in different normal adult tissues, *ACTB* had the lowest comprehensive analysis ranking values of RT-qPCR and the highest variance, which was not included in the candidate reference gene list. *TBP* and *ATPase B* were more stable with relatively high rankings of stability by RT-qPCR compared with other traditional reference genes (Fig. 3B), which is expected because the two genes are candidate reference genes that pass the criteria and have lower CV values in five commonly used reference genes (Table 3). The results of the comprehensive analysis in early development and normal adult tissues are in accordance with those of previous studies in *U. unicinctus* [52]. During sulfide stress in the hindgut, *ACTB* was the most unstable in the comprehensive RT-qPCR results (Fig. 3C). However, there was also some inconsistency between the results of RT-qPCR and transcriptome data analysis. For example, *GAPDH*, which ranked second after *STX12* in the RT-qPCR results during early development (Fig. 3A), was not included in the candidate reference gene list with a 1.23 of SD log_2_(TPM) value by transcriptome data analysis. We deduced that the inconsistent phenomenon may result from the difference in the samples between the transcriptome data and RT-qPCR.

### Relationship of gene expression level between transcriptome data and RT-qPCR

**Fig. 4:**
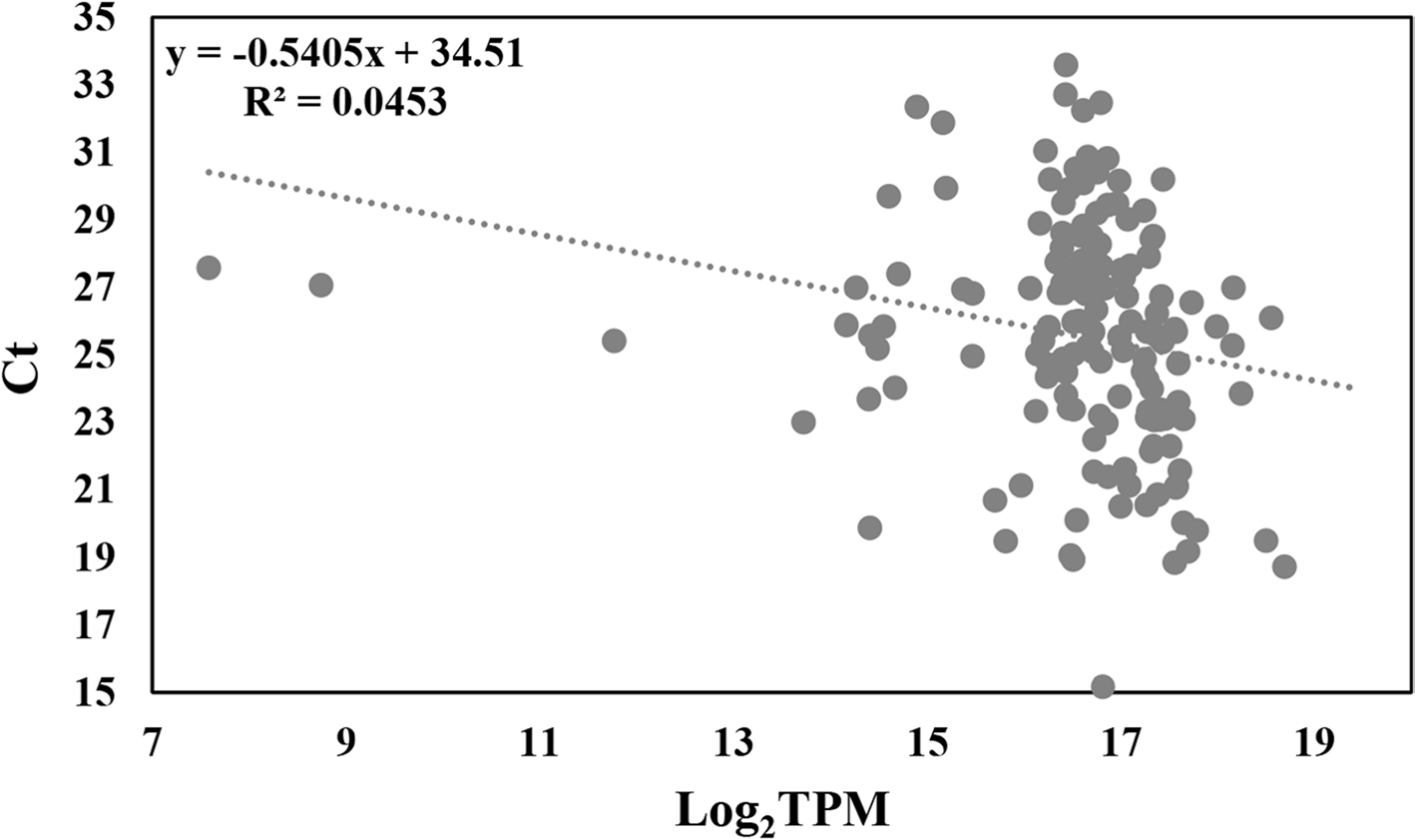
The correlation of gene expression between log_2_TPM of transcriptome data and Ct values of RT-qPCR

Previous studies have suggested that there is a high correlation between RNA-Seq data and Ct value of RT-qPCR [11]. Therefore, we assessed the relationship between the TPM values of the transcriptome data and RT-qPCR data. As shown in Fig. 4, there was a significant negative correlation between log_2_(TPM) and Ct values (*R*^2^ = 0.0453, *P* < 0.0001), with the formula Ct =  − 0.5405 log_2_(TPM) + 34.51. This formula will contribute to the estimation the Ct value based on transcriptome data without executing the RT-qPCR assay, and will be conducive to our further research.

## Conclusions

In this study, we identified candidate reference genes for embryos and larvae of early developmental stages, normal adult tissues, and the hindgut under sulfide stress based on transcriptome data from *U. unicinctus*. We then validated of the candidate reference genes by RT-qPCR using four methods (geNorm, NormFinder, BestKeeper, and ∆Ct) and compared the stability between the candidate reference genes and commonly used reference genes. The results showed that *STX12*, *EHMT1*, and *LYAG* are the most stable candidate reference genes in early development, normal adult tissues, and the hindgut under sulfide stress, respectively. Our study indicates that transcriptome analysis approaches have great potential to discover novel stable reference genes and will contribute to future gene expression level research in *U. unicinctus*.

## Materials and methods

### Animals materials and treatments

Adult *U. unicinctus* were collected from the intertidal zone along the coast of Yantai city, China. They were maintained in aerated seawater (19℃, pH8.0, salinity 30 PSU) and raised with *Chaetoceros muelleri*, *Chlorella vulgaris*, and *Platymonas helgolandica*.

We selected three healthy adult worms and dissected six tissues, including the body wall, coelomic fluid, foregut, mid-gut, hindgut and anal sac from each individual in phosphate-buffered saline (PBS, pH7.4). After dissection, the tissues were immediately frozen in liquid nitrogen and stored at -80℃.

Sexually mature individuals were selected and dissected to acquire mature ova and sperm from nephridia (gonoducts) during the spawning season. Sperm and ova were then mixed for artificial insemination at ratio of 10:1. Fertilized eggs were reared in filtered seawater (FSW) (17℃, pH 7.9, salinity 30). Embryos and larvae from ten developmental stages, including early cells (EC, 2 cells, 4 cells, and 8 cells), multiple cell (MC, generally more than 32 cells), blastulae (BL), gastrulae (GA), early trochophore larva (ET, 1 d post fertilization, dpf), mid-trochophore larva (MT, 2 dpf), late-trochophore larva (LT, 25 dpf), early segmentation larva (ES, 30 dpf), segmentation larvae (SL, 35 dpf) and worm-shaped larvae (WL, 42 dpf), were collected, frozen immediately in liquid nitrogen and then stored at -80℃ for total RNA extraction. Three biological replicates were prepared for each developmental stage.

The experimental system and sulfide treatment were conducted as described previously [45]. We prepared three aquariums containing 30 L of seawater and sealed them with a cling film. Six individuals were randomly selected for placement in each aquarium. The sulfide concentration in seawater was maintained at 50 μM (equivalent to moderately polluted sediment that *U. unicinctus* can live normally) by adding the sulfide stock solution (10 mM Na_2_S, pH 8.0) at 2 h interval, and detected by the methylene blue method [41]. The hindguts of three individuals from each aquarium were dissected 0 (control), 6, 24, and 48 h after sulfide treatment. The hindgut was immediately frozen in liquid nitrogen and stored at − 80 °C.

### Transcriptome datasets

Transcriptome data of *U. unicinctus* embryos and larvae at various developmental periods were obtained from the NCBI Sequence Read Archive (SRA) database under the accession numbers PRJNA485379 and PRJNA394029, mainly including the following stages, EC: Early cells; MC: Multicellular; BL: Blastula; GA: Gastrula; ET: Early-trochophore; MT: Mid-trochophore; LT: Late-trochophore; SL: Segmentation larva and WL: Worm-shaped larva. The transcriptome data of the normal adult tissues were also obtained from the NCBI SRA database under the accession number PRJNA917787, which mainly included the body wall, coelomic fluid, foregut, mid-gut, hindgut, and anal sac. Transcriptome data of the sulfide stress hindgut samples (50 μM for 0, 6, 24, and 48 h) were also obtained from the NCBI SRA database under the accession number PRJNA752504.

### Identification of reference genes based on transcriptome data

Reference genes for RT-qPCR were selected using the coefficient of variation (CV) method as previously described [11]. TPM values were used to measure gene expression levels and averaged for subsequent analyses of biological replicates. Firs, genes with log_2_(TPM) values less than or equal to 5 were excluded, because these low-expression genes would lead to poor RT-qPCR results, which makes it difficult to detect and quantify their expression. CV values were calculated using the formula CV = standard deviation (SD) of log_2_(TPM) / average log_2_(TPM) (mean). Calculations for the mean, SD, and CV were implemented in Microsoft Excel. Candidate reference genes with low variances were required, with SD values lower than 1. Therefore, a CV cut-off of 0.2 for stable genes was adopted, which was the cut-off for stable expression across heterogeneous genes.

### Functional enrichment analysis

To further understand the functions of the selected candidate reference genes, Gene Ontology (GO) terms and Kyoto Encyclopedia of Genes and Genomes (KEGG) enrichment analysis were performed. The Swiss-Prot Blast results for all genes and the results of reference genes were imported into the online software OmicShare Tools (https://www.omicshare.com/tools/home/index/index.html), and the GO and KEGG enrichment analysis was completed using the Bioinformatics Cloud Tool Platform [78–80].

### RNA isolation and cDNA synthesis

Total RNA was extracted from the stored different samples using TRIzol reagent (Invitrogen, Carlsbad, CA, USA), according to the manufacturer’s instructions. The RNA quality was assessed using NanoDrop 2000 (Thermo Scientific, Wilmington, DE, USA) and agarose gel electrophoresis. Then, the cDNA template was prepared using a PrimeScript™ RT reagent kit with gDNA Eraser (TaKaRa, Dalian, China) following the manufacturing’s instruction, and diluted with distilled water (1:10) for subsequent experiments.

### Validation of the reference gene expression stability by RT-qPCR assay

Ten genes, consisting of five novel candidates and five commonly used reference genes, were chosen for RT-qPCR validation in early development, normal adult tissues, and the hindgut under sulfide stress. Primers were designed using Primer Primier software (5.0) and the primer sequences are listed in Additional file 1: Table S1.

RT-qPCR was performed on Light Cycler 480 system (Roche, Basel, Switzerland) using SYBR Premix Ex TaqTM (TaKaRa, Dalian, China). All reactions were carried out with three sample replicates and three technical replicates, and all RT-qPCR assays were validated in compliance with “MIQE guidelines” [81].

Four statistical approaches, geNorm (https://genorm.cmgg.be/) [57], NormFinder (http://moma.dk/) [58], BestKeeper (www.gene-quantification.com/bestkeeper.html) [59], and ∆Ct method [60], were applied to estimate the expression stability of the reference genes. The final ranking of gene expression stability was determined by calculating the geometric mean values of the results acquired using the four approaches.

### Statistical analysis

Statistical analysis of the correlation between FPKM and Ct values was performed using one-way analysis of variance (one-way ANOVA). Statistical significance was set at *P* < 0.05.

## Supplementary Information

**Additional file 1:**  **Table S1.** Information of primers of reference genes in RT-qPCR validation.

## Data Availability

The datasets analysed during the current study are available in the NCBI SRA repository (PRJNA485379, PRJNA394029, PRJNA752504 and PRJNA917787).

## Abbreviations

RT-qPCR: Reverse transcription quantitative PCR
GAPDH: Glyceraldehyde-3-phosphate dehydrogenase
EF-1-α: Elongation factor 1 alpha
TBP: TATA-box-binding protein
TUB: Beta-tublin
eIF3: Eukaryotic initiation factors 3
ATPase B: Mitochondrial adenosine triphosphate synthase subunit b
ACTB: Beta-actin
FPKM: Fragments per kilobase million
TPM: Transcripts per million
RPKM: Reads per kilobase million
PBS: Phosphate buffer saline
FSW: Filtered sea water
EC: Early cells
MC: Multiple cell
BL: Blastulae
GA: Gastrulae
ET: Early-trochophore larva
MT: Mid-trochophore larvae
LT: Late-trochophore larvae
ES: Early-segmentation larvae
SL: Segmentation larvae
WL: Worm-shaped larvae
CV: Coefficient of variation
GO: Gene Ontology
KEGG: Kyoto Encyclopedia of Genes and Genomes
FXRD1: FAD-dependent oxidoreductase domain-containing protein 1
CAPR1: Caprin-1
NSMA: Neutral sphingomyelinase
IF2M: Translation initiation factor IF-2
STX12: Syntaxin-12
UBE4A: Ubiquitin conjugation factor E4 A
OGT1: UDP-N-acetylglucosamine–peptide N-acetylglucosaminyltransferase 110 kDa subunit
GGA3: ADP-ribosylation factor-binding protein
EHMT1: Histone-lysine N-methyltransferase
TRA2B: Transformer-2 protein homolog beta
CNOT1: CCR4-NOT transcription complex subunit 1
CLH1: Clathrin heavy chain 1
EXOC6: Exocyst complex component 6
LYAG: Lysosomal alpha-glucosidase
TRFM: Melanotransferrin

## References

[1] Bustin SA, Benes V, Nolan T, Pfaffl MW (2005). Quantitative real-time RT-PCR–a perspective. J Mol Endocrinol.

[2] Kubista M, Andrade JM, Bengtsson M, Forootan A, Jonák J, Lind K, Sindelka R, Sjöback R, Sjögreen B, Strömbom L (2006). The real-time polymerase chain reaction. Mol Aspects Med.

[3] Green MR, Sambrook J. Quantification of RNA by Real-Time Reverse Transcription-Polymerase Chain Reaction (RT-PCR). Cold Spring Harb Protoc. 2018;2018(10):847-56. 10.1101/pdb.prot095042 30275077

[4] Li Z, Li X, Zhang Q, Yuan L, Zhou X (2020). Reference gene selection for transcriptional profiling in *Cryptocercus punctulatus*, an evolutionary link between Isoptera and Blattodea. Sci Rep.

[5] Harshitha R, Arunraj DR (2021). Real-time quantitative PCR: A tool for absolute and relative quantification. Biochem Mol Biol Educ.

[6] Zhang Y, Zhang Z, Ren M, Liu X, Zhou X, Yang J (2022). Selection of Reference Genes for RT-qPCR Analysis in the Hawthorn Spider Mite, *Amphitetranychus viennensis* (Acarina: Tetranychidae), Under Acaricide Treatments. J Econ Entomol.

[7] da Conceição BL, Gonçalves BÔP, Coelho PL, da Silva Filho AL, Silva LM (2022). Identification of best housekeeping genes for the normalization of RT-qPCR in human cell lines. Acta Histochem.

[8] Li J, Fu N, Ren L, Luo Y (2022). Identification and Validation of Reference Genes for Gene Expression Analysis in *Monochamus saltuarius* Under *Bursaphelenchus xylophilus* Treatment. Front Physiol.

[9] Song J, Cho J, Park J, Hwang JH (2022). Identification and validation of stable reference genes for quantitative real time PCR in different minipig tissues at developmental stages. BMC Genomics.

[10] Gao D, Kong F, Sun P, Bi G, Mao Y (2018). Transcriptome-wide identification of optimal reference genes for expression analysis of *Pyropia yezoensis* responses to abiotic stress. BMC Genomics.

[11] Li Y, Zhang L, Li R, Zhang M, Li Y, Wang H, Wang S, Bao Z (2019). Systematic identification and validation of the reference genes from 60 RNA-Seq libraries in the scallop *Mizuhopecten yessoensis*. BMC Genomics.

[12] Wang X, Wu Z, Bao W, Hu H, Chen M, Chai T, Wang H (2019). Identification and evaluation of reference genes for quantitative real-time PCR analysis in *Polygonum cuspidatum* based on transcriptome data. BMC Plant Biol.

[13] Yi S, Lin Q, Zhang X, Wang J, Miao Y, Tan N (2020). Selection and Validation of Appropriate Reference Genes for Quantitative RT-PCR Analysis in *Rubia yunnanensis* Diels Based on Transcriptome Data. Biomed Res Int.

[14] Czechowski T, Stitt M, Altmann T, Udvardi MK, Scheible W-R (2005). Genome-Wide Identification and Testing of Superior Reference Genes for Transcript Normalization in Arabidopsis. Plant Physiol.

[15] Gabrielsson BG, Olofsson LE, Sjögren A, Jernås M, Elander A, Lönn M, Rudemo M, Carlsson LM (2005). Evaluation of reference genes for studies of gene expression in human adipose tissue. Obes Res.

[16] Gong L, Yang Y, Chen Y, Shi J, Song Y, Zhang H (2016). *LbCML38* and *LbRH52*, two reference genes derived from RNA-Seq data suitable for assessing gene expression in *Lycium barbarum* L. Sci Rep.

[17] Guo H, Jiang L, Xia Q (2016). Selection of reference genes for analysis of stress-responsive genes after challenge with viruses and temperature changes in the silkworm *Bombyx mori*. Mol Genet Genomics.

[18] Hu Y, Xie S, Yao J (2016). Identification of Novel Reference Genes Suitable for qRT-PCR Normalization with Respect to the Zebrafish Developmental Stage. PLoS ONE.

[19] Kudo T, Sasaki Y, Terashima S, Matsuda-Imai N, Takano T, Saito M, Kanno M, Ozaki S, Suwabe K, Suzuki G (2016). Identification of reference genes for quantitative expression analysis using large-scale RNA-seq data of *Arabidopsis thaliana* and model crop plants. Genes Genet Syst.

[20] Vieira A, Cabral A, Fino J, Azinheira HG, Loureiro A, Talhinhas P, Pires AS, Varzea V, Moncada P, Oliveira H (2016). Comparative Validation of Conventional and RNA-Seq Data-Derived Reference Genes for qPCR Expression Studies of *Colletotrichum kahawae*. PLoS ONE.

[21] Zhou Z, Cong P, Tian Y, Zhu Y (2017). Using RNA-seq data to select reference genes for normalizing gene expression in apple roots. PLoS ONE.

[22] Mughal BB, Leemans M, Spirhanzlova P, Demeneix B, Fini J-B (2018). Reference gene identification and validation for quantitative real-time PCR studies in developing *Xenopus laevis*. Sci Rep.

[23] Liu F, Li X, Ji Y, Liu C, Sun T, Zhao Y, Nicolae CG, Dediu L (2019). THE BIOLOGICAL CHARACTERISTICS AND UTILIZATION OF *Urechis unicinctus*. AgroLife Sci J.

[24] Tan X, Wang YC, Sun QY, Peng A, Chen DY, Tang YZ (2005). Effects of MAP kinase pathway and other factors on meiosis of *Urechis unicinctus* eggs. Mol Reprod Dev.

[25] Qin Z, Zhang Y, Mu H, Zhang Z, Qiu JW (2018). The sperm proteome of the echiuran *Urechis unicinctus* (Annelida, Echiura). Proteomics.

[26] Han Y-H, Ryu K-B, Medina Jiménez BI, Kim J, Lee H-Y, Cho S-J (2020). Muscular Development in *Urechis unicinctus* (Echiura, Annelida). Int J Mol Sci.

[27] Fujiwara A, Tazawa E, Hino A, Asami K, Yasumasu I (1986). Respiration in Eggs of the Echiuroid, *Urechis unicinctus*, Before and After Fertilization: echiuroid eggs/fertilization/respiration/redox dyes/uncoupler of oxidative phosphorylation. Dev Growth Differ.

[28] Kojima MK (1959). On the vitally stainable granules in the egg of the echiuroid Urechis unicinctus. Embryologia.

[29] Hou X, Qin Z, Wei M, Fu Z, Liu R, Lu L, Bai S, Ma Y, Zhang Z (2020). Identification of the neuropeptide precursor genes potentially involved in the larval settlement in the Echiuran worm *Urechis unicinctus*. BMC Genomics.

[30] Bai S, Fan S, Liu D, Zhang Z, Zhang Z (2022). Identification and expression analysis of receptors that mediate MIP regulating larval settlement in *Urechis unicinctus*. Comp Biochem Physiol B: Biochem Mol Biol.

[31] Lu L, Zhang Z, Zheng Q, Chen Z, Bai S, Zhang Z (2022). Expression Characteristics and Potential Function of Neuropeptide MIP in Larval Settlement of the Echiuran Worm *Urechis unicinctus*. J Ocean Univ China.

[32] Wang J, Zhang L, Lian S, Qin Z, Zhu X, Dai X, Huang Z, Ke C, Zhou Z, Wei J, Liu P, Hu N, Zeng Q, Dong B, Dong Y, Kong D, Zhang Z, Liu S, Xia Y, Li Y, Zhao L, Xing Q, Huang X, Hu X, Bao Z, Wang S (2020). Evolutionary transcriptomics of metazoan biphasic life cycle supports a single intercalation origin of metazoan larvae. Nat Ecol Evol.

[33] Wei M, Qin Z, Kong D, Liu D, Zheng Q, Bai S, Zhang Z, Ma Y (1982). Echiuran Hox genes provide new insights into the correspondence between Hox subcluster organization and collinearity pattern. Proc Biol Sci.

[34] Ma YB, Zhang ZF, Shao MY, Kang KH, Tan Z, Li JL (2011). Sulfide:quinone oxidoreductase from echiuran worm *Urechis unicinctus*. Mar Biotechnol.

[35] Zandvakili A, Gebelein B (2016). Mechanisms of Specificity for Hox Factor Activity. J Dev Biol.

[36] Ma Y-B, Zhang Z-F, Shao M-Y, Kang K-H, Shi X-L, Dong Y-P, Li J-L (2012). Response of sulfide: quinone oxidoreductase to sulfide exposure in the echiuran worm *Urechis unicinctus*. Mar Biotechnol.

[37] Ma Y-B, Zhang Z-F, Shao M-Y, Kang K-H, Zhang L-T, Shi X-L, Dong Y-P (2012). Function of the anal sacs and mid-gut in mitochondrial sulphide metabolism in the echiuran worm *Urechis unicinctus*. Mar Biol Res.

[38] Zhang L, Liu X, Liu J, Zhang Z (2013). Characteristics and function of sulfur dioxygenase in echiuran worm *Urechis unicinctus*. PLoS ONE.

[39] Liu X, Qin Z, Li X, Ma X, Gao B, Zhang Z (2016). NF1, Sp1 and HSF1 are synergistically involved in sulfide-induced sqr activation in echiuran worm *Urechis unicinctus*. Aquat Toxicol.

[40] Liu X, Zhang Z, Ma X, Li X, Zhou D, Gao B, Bai Y (2016). Sulfide exposure results in enhanced sqr transcription through upregulating the expression and activation of HSF1 in echiuran worm *Urechis unicinctus*. Aquat Toxicol.

[41] Zhang L, Liu X, Qin Z, Liu J, Zhang Z (2016). Expression characteristics of sulfur dioxygenase and its function adaption to sulfide in echiuran worm *Urechis unicinctus*. Gene.

[42] Li X, Liu X, Qin Z, Wei M, Hou X, Zhang T, Zhang Z (2018). A novel transcription factor Rwdd1 and its SUMOylation inhibit the expression of sqr, a key gene of mitochondrial sulfide metabolism in *Urechis unicinctus*. Aquat Toxicol.

[43] Zhang L, Zhang Z (2019). The response of sulfur dioxygenase to sulfide in the body wall of *Urechis unincinctus*. Peer J.

[44] Zhang T, Qin Z, Liu D, Wei M, Fu Z, Wang Q, Ma Y, Zhang Z (2021). A novel transcription factor MRPS27 up-regulates the expression of sqr, a key gene of mitochondrial sulfide metabolism in echiuran worm *Urechis unicinctus*. Comp Biochem Physiol C: Toxicol Pharmacol.

[45] Liu D, Qin Z, Wei M, Kong D, Zheng Q, Bai S, Lin S, Zhang Z, Ma Y (2022). Genome-Wide Analyses of Heat Shock Protein Superfamily Provide New Insights on Adaptation to Sulfide-Rich Environments in *Urechis unicinctus* (Annelida, Echiura). Int J Mol Sci.

[46] Hou X, Wei M, Li Q, Zhang T, Zhou D, Kong D, Xie Y, Qin Z, Zhang Z (2019). Transcriptome Analysis of Larval Segment Formation and Secondary Loss in the Echiuran Worm *Urechis unicinctus*. Int J Mol Sci.

[47] Liu X, Zhang L, Zhang Z, Ma X, Liu J (2015). Transcriptional response to sulfide in the Echiuran Worm *Urechis unicinctus* by digital gene expression analysis. BMC Genomics.

[48] Ma X, Liu X, Zhou D, Bai Y, Gao B, Zhang Z, Qin Z (2016). The NF-κB pathway participates in the response to sulfide stress in *Urechis unicinctus*. Fish Shellfish Immunol.

[49] Shi X, Shao M, Zhang L, Ma Y, Zhang Z (2012). Screening of genes related to sulfide metabolism in *Urechis unicinctus* (Echiura, Urechidae) using suppression subtractive hybridization and cDNA microarray analysis. Comp Biochem Physiol D: Genomics Proteomics.

[50] Huang J, Zhang L, Li J, Shi X, Zhang Z (2013). Proposed function of alternative oxidase in mitochondrial sulphide oxidation detoxification in the Echiuran worm Urechis unicinctus. J Mar Biolog.

[51] Oh HY, Kim CH, Go HJ, Park NG (2018). Isolation of an invertebrate-type lysozyme from the nephridia of the echiura, *Urechis unicinctus*, and its recombinant production and activities. Fish Shellfish Immunol.

[52] Bai Y, Zhou D, Wei M, Xie Y, Gao B, Qin Z, Zhang Z (2018). Identification of reference genes for normalizing quantitative real-time PCR in *Urechis unicinctus*. J Ocean Univ China.

[53] Wei M, Lu L, Wang Q, Kong D, Zhang T, Qin Z, Zhang Z (2019). Evaluation of suitable reference genes for normalization of RT-qPCR in Echiura (*Urechis unicinctus*) during developmental process. Russ J Mar Biol.

[54] Park C, Han YH, Lee SG, Ry KB, Oh J, Kern EMA, Park JK, Cho SJ (2018). The developmental transcriptome atlas of the spoon worm *Urechis unicinctus* (Echiurida: Annelida). Gigascience.

[55] Stanton KA, Edger PP, Puzey JR, Kinser T, Cheng P, Vernon DM, Forsthoefel NR, Cooley AM (2017). A Whole-Transcriptome Approach to Evaluating Reference Genes for Quantitative Gene Expression Studies: A Case Study in *Mimulus*.. G3 (Bethesda).

[56] Dos Santos KCG, Desgagné-Penix I, Germain H (2020). Custom selected reference genes outperform pre-defined reference genes in transcriptomic analysis. BMC Genomics.

[57] Vandesompele J, De Preter K, Pattyn F, Poppe B, Van Roy N, De Paepe A, Speleman F (2002). Accurate normalization of real-time quantitative RT-PCR data by geometric averaging of multiple internal control genes. Genome Biol.

[58] Andersen CL, Jensen JL, Ørntoft TF (2004). Normalization of real-time quantitative reverse transcription-PCR data: a model-based variance estimation approach to identify genes suited for normalization, applied to bladder and colon cancer data sets. Can Res.

[59] Pfaffl MW, Tichopad A, Prgomet C, Neuvians TP (2004). Determination of stable housekeeping genes, differentially regulated target genes and sample integrity: BestKeeper–Excel-based tool using pair-wise correlations. Biotech Lett.

[60] Silver N, Best S, Jiang J, Thein SL (2006). Selection of housekeeping genes for gene expression studies in human reticulocytes using real-time PCR. BMC Mol Biol.

[61] Tang BL, Tan AE, Lim LK, Lee SS, Low DY, Hong W (1998). Syntaxin 12, a member of the syntaxin family localized to the endosome. J Biol Chem.

[62] Minakami R, Kato A, Sugiyama H (2000). Interaction of Vesl-1L/Homer 1c with syntaxin 13. Biochem Biophys Res Commun.

[63] Goh CS, Cohen FE (2002). Co-evolutionary analysis reveals insights into protein-protein interactions. J Mol Biol.

[64] Subramaniam VN, Loh E, Horstmann H, Habermann A, Xu Y, Coe J, Griffiths G, Hong W (2000). Preferential association of syntaxin 8 with the early endosome. J Cell Sci.

[65] Das J (2020). SNARE Complex-Associated Proteins and Alcohol. Alcohol Clin Exp Res.

[66] Prekeris R, Klumperman J, Chen YA, Scheller RH (1998). Syntaxin 13 mediates cycling of plasma membrane proteins via tubulovesicular recycling endosomes. J Cell Biol.

[67] Battisti V, Pontis J, Boyarchuk E, Fritsch L, Robin P, Ait-Si-Ali S, Joliot V (2016). Unexpected Distinct Roles of the Related Histone H3 Lysine 9 Methyltransferases G9a and G9a-Like Protein in Myoblasts. J Mol Biol.

[68] Pless O, Kowenz-Leutz E, Knoblich M, Lausen J, Beyermann M, Walsh MJ, Leutz A (2008). G9a-mediated lysine methylation alters the function of CCAAT/enhancer-binding protein-beta. J Biol Chem.

[69] Karl M, Sommer C, Gabriel CH, Hecklau K, Venzke M, Hennig AF, Radbruch A, Selbach M, Baumgrass R (2019). Recruitment of Histone Methyltransferase Ehmt1 to Foxp3 TSDR Counteracts Differentiation of Induced Regulatory T Cells. J Mol Biol.

[70] Kerchner KM, Mou TC, Sun Y, Rusnac DV, Sprang SR, Briknarová K (2021). The structure of the cysteine-rich region from human histone-lysine N-methyltransferase EHMT2 (G9a). J Struct Biol-X.

[71] Pareek C, Michno J, Smoczynski R, Tyburski J, Golebiewski M, Piechocki K, Wimmers K (2013). Identification of predicted genes expressed differentially in pituitary gland tissue of young growing bulls revealed by the cDNA-AFLP technique. Czeh J Anim Sci.

[72] Collins R, Cheng X (2010). A case study in cross-talk: the histone lysine methyltransferases G9a and GLP. Nucleic Acids Res.

[73] Kato A, Nakagome I, Hata M, Nash RJ, Fleet GWJ, Natori Y, Yoshimura Y, Adachi I, Hirono S (2020). Strategy for Designing Selective Lysosomal Acid α-Glucosidase Inhibitors: Binding Orientation and Influence on Selectivity. Molecules.

[74] Hamura R, Shirai Y, Shimada Y, Saito N, Taniai T, Horiuchi T, Takada N, Kanegae Y, Ikegami T, Ohashi T, Yanaga K (2021). Suppression of lysosomal acid alpha-glucosidase impacts the modulation of transcription factor EB translocation in pancreatic cancer. Cancer Sci.

[75] Hoefsloot LH, Hoogeveen-Westerveld M, Kroos M, Van Beeumen J, Reuser AJ, Oostra B (1988). Primary structure and processing of lysosomal alpha-glucosidase; homology with the intestinal sucrase-isomaltase complex. EMBO J.

[76] Cagin U, Puzzo F, Gomez MJ, Moya-Nilges M, Sellier P, Abad C, Van Wittenberghe L, Daniele N, Guerchet N, Gjata B, Collaud F, Charles S, Sola MS, Boyer O, Krijnse-locker J, Ronzitti G, Colella P, Mingozzi F (2020). Rescue of Advanced Pompe Disease in Mice with Hepatic Expression of Secretable Acid α-Glucosidase. Mol Ther.

[77] Le Chevalier P, Sellos D, Van Wormhoudt A (2000). Molecular cloning of a cDNA encoding alpha-glucosidase in the digestive gland of the shrimp, *Litopenaeus vannamei*. Cell Mol Life Sci.

[78] Kanehisa M, Goto S (2000). KEGG: Kyoto Encyclopedia of Genes and Genomes. Nucleic Acids Resarch.

[79] Kanehisa M (2019). Toward understanding the origin and evolution of cellular organisms. Protein Sci.

[80] Kanehisa M, Furumichi M, Sato Y, Kawashima M, Ishiguro-Watanabe M (2023). KEGG for taxonomy-based analysis of pathways and genomes. Nucleic Acids Res.

[81] Bustin SA, Benes V, Garson JA, Hellemans J, Huggett J, Kubista M, Mueller R, Nolan T, Pfaffl MW, Shipley GL, Vandesompele J, Wittwer CT (2009). The MIQE guidelines: minimum information for publication of quantitative real-time PCR experiments. Clin Chem.

